# Intra- and interpersonal emotion regulation and adjustment symptoms in couples: The role of co-brooding and co-reappraisal

**DOI:** 10.1186/s40359-016-0159-7

**Published:** 2016-10-28

**Authors:** Andrea B. Horn, Andreas Maercker

**Affiliations:** 1 Psychopathology and Clinical Intervention Unit, University of Zurich, Zürich, Switzerland; 2 “Dynamics of Healthy Aging”, University Research Priority Program (URPP), Andreasstrasse 15/2, 8050 Zürich, Switzerland

**Keywords:** Psycho-social adjustment to stress, Adjustment disorder, Emotion regulation, Interpersonal emotion regulation, Couples, Rumination, Reappraisal, Co-brooding, Co-reappraisal

## Abstract

**Background:**

Adult emotion regulation is not only occurring within the person but includes strategies that happen in social interactions and that are framed as co-regulating. The current study investigates the role of the interpersonal emotion regulation strategies of co-reappraisal and co-brooding in couples for adjustment disorder symptoms as the disorder will be outlined in the International Classification of Diseases-11 (ICD-11).

**Methods:**

Couples registered together in an online questionnaire study reporting whether or not they are adjusting to a major stressor that is psychologically challenging to them. In total, one hundred and forty-six participants (*N* = 73 male; *N* = 73 female) reported having experienced a major stressor in the last 12 months and were thus be identified as at risk for adjustment disorder. Those individuals at risk were assessed for adjustment disorder and depressive symptoms; intra- and interpersonal emotion regulation (co-/brooding, co-/reappraisal) were assessed not only in the individual at risk but also in the romantic partner.

**Results:**

Regression-based dyadic analyses revealed that above and beyond intrapersonal emotion regulation, interpersonal co-brooding and for the female participants also co-reappraisal were significantly associated with symptoms of adjustment disorder and depression, standardized betas varied between .24 and .36, suggesting medium effect sizes. An association with the female partner’s tendency to reappraise with fewer symptoms in the male partner at risk for adjustment disorder could also be observed.

**Conclusions:**

Co-brooding and co-reappraisal represent emotion regulation strategies that happen in social interaction and seem to play a relevant role in the context of adjustment disorders above and beyond the commonly assessed intrapersonal emotion regulation strategies.

**Electronic supplementary material:**

The online version of this article (doi:10.1186/s40359-016-0159-7) contains supplementary material, which is available to authorized users.

## Background

In the last decade, the notion that emotion regulation strategies are deployed in solitude inside an individual like a “lone man fighting against the elements” has been challenged [1]. Emotions tend to be elicited but also regulated in the social context [2]. Recent approaches imply it might be the rule and not the exception that the regulation of emotions occurs in the social context not only in childhood, as is often assumed, but throughout the life span [2–5]. It has been suggested that interpersonal processes can regulate emotion on direct pathways as they have been introduced in models of intrapersonal emotion regulation. As an example, cognitive change, attention deployment or changes of emotional expression can happen during interactive processes [6]. Furthermore, an indirect socio-affective pathway via changes in relationship quality that in turn affect emotional states can be assumed [7, 8]. In other words, when emotion regulation happens in interaction additionally to the known intrapersonal changes, social processes linked to affect may be altered which represents a genuine interpersonal level of regulation. However, research focusing on interpersonal strategies of emotion regulation has been sparse, at least compared to research with an intrapersonal focus [9]. Increasing attention has been given to the fact that interpersonal emotion regulation is important in the context of mental health, for example in depression [10] and anxiety disorders [11]. Regarding stress-and trauma-response disorders, A Maercker and AB Horn [12] have introduced the socio-interpersonal model as a conceptual framework integrating a variety of findings from the literature. In this model, the importance of the socio-interpersonal context is accentuated not only in the aftermath of traumatic events but also in adjusting to severe stressful events that may provoke adjustment disorder. In this paper, the role of inter- and intrapersonal emotion regulation at the level of romantic relationships for adjustment disorder symptoms is the target of investigation. We apply a recent reformulation of adjustment disorder that will appear in version 11 of the International Classification of Diseases (ICD-11) [13] that focuses on two symptom clusters: preoccupation (recurrent and distressing thoughts about stressors) and failure to adapt (symptoms interfering with daily functioning). Following the stress-response view of adjustment disorder all symptoms are clearly assessed in the context the stressful event that initiated the adjustment problems. This is important to note as this is a promising way to distinguish adjustment problems from general depression.

## The interpersonal view on emotion regulation

Emotion regulation has been conceptualized as a process through which affective reactions are modulated [14]. In this study, interpersonal emotion regulation is defined as emotion regulation sensu JJ Gross and RA Thompson [14] that happens in interaction. As other authors suggest, in adulthood interpersonal emotion regulation with a romantic partner is of particular importance [2, 15, 16]. This is to be expected as the romantic relationship implies the highest level of psychological intimacy in adulthood [17]. In this couple study, the association between intra- and interpersonal emotion regulation strategies on adjustment disorder and depressive symptoms is investigated on a dyadic level, taking into account the potential actor and partner effects of emotion regulation strategies. The main question is whether the newly introduced interpersonal emotion regulation strategies of co-reappraisal and co-brooding will predict adjustment problems above and beyond the established parallel intrapersonal strategies of reappraisal and brooding.

In the growing literature on emotion regulation strategies, two strategies have proven to be of major importance: rumination as a maladaptive emotion regulation strategy and reappraisal as an adaptive emotion regulation strategy [18]. However, it is to assume that these emotion regulation strategies are not limited to intrapersonal processes but might also be applied in interaction in the couple. In the following a parallel view of intra- and interpersonal emotion regulation strategies of reappraisal and ruminative brooding is introduced.

### Reappraisal and co-reappraisal

Reappraisal is defined as a change in the appraisal of a situation in order to decrease its negative emotional impact [19]. It is one of the most researched adaptive coping strategies that has adaptive effects on the regulation of negative emotions if experimentally induced [20] as well as when assessed by self report in the context of mental health [21, 22]. The notion that changing one’s view on an issue that elicits emotion not only happens in introspective reflection but tends to happen in conversation with an interaction partner is plausible. This is in line with Rime’s [1] notion that the motives behind social sharing, that is, the sharing of emotional content with others, are clarification, cognitive restructuring, and meaning finding. Co-reappraisal is defined as changing a situation’s meaning in a way that alters its emotional impact *in interaction* (see [14]. Accordingly, co-reappraisal in this study is measured as the intent to do so in interactions with the romantic partner. In general, as interactive emotion regulation affects the mental and social realities of the regulating individual, it is supposed to be particularly broad in its effects.

### Ruminative brooding and co-brooding

Rumination is a maladaptive coping strategy and a risk factor for depression [23] that has been proven to be associated with a wide array of negative outcomes [24]. Recently, it has been suggested that different ruminative components can be separated: reflection has been defined as an adaptive component of negative self-focus, as opposed to the maladaptive component which has been labeled “ruminative brooding” [25]. While adaptive ruminative reflection is characterized by purposefully engaging in problem solving-oriented cognitive-affective processing, ruminative brooding is the passive comparison of one’s current situation with some unachieved standards characterized by cognitive superficiality and avoidance [26].

The notion that rumination might happen in interactions reflecting a repetitive focus on negative content in conversations with others who are close has been introduced as co-rumination [27]. Co-rumination is a risk factor for the onset of a depressive episode in adolescence [28]. Recently, the parallel factor structure of co-rumination to intrapersonal rumination has been demonstrated in a study assessing co-rumination in children; the factors co-brooding and co-reflection were detected reassembling the findings of a brooding and a reflection factor in intrapersonal rumination [29]. In the current study, co-brooding is referred to as the merely maladaptive component of co-rumination. Parallel to brooding, co-brooding is characterized by a passive repetitive focus on negative content that is unwanted, rigid and perceived as unpleasant. It lacks the possibility to reflect upon the content, process it in a constructive way, and possibly reappraise it. Interactive co-brooding in communication is supposed to be associated with the difficulty of the conversation partner to react in a responsive way. Thus, instead of perceiving responsiveness—being understood, cared for, and validated [30]—the co-brooding person might feel less understood and less supported, the relationship quality suffers. Thus, even though it is also a way of sharing negative content, it can be distinguished from adaptive forms of social sharing which are supposed to lead to better relationship quality and less loneliness after an unpleasant situation [1]. Co-rumination has been shown to be an independent risk factor predicting depression above and beyond intrapersonal rumination as a coping style [28]. Accordingly, co-brooding is thought to be an interactive form of brooding that is distinct from intrapersonal brooding.

### Aim of the study

The socio-interpersonal perspective on stress-response suggests that interpersonal processes are of particular importance. The aim of the study was to investigate interpersonal emotion regulation as a fundamental pathway through which interpersonal processes might shape adjustment. It was predicted that genuine interpersonal emotion regulation strategies co-reappraisal and co-brooding are associated with adjustment disorder symptoms after a major stressor. Furthermore, it was expected that co-brooding and co-reappraisal in couples predict symptoms of adjustment disorder above and beyond the established intrapersonal emotion regulation strategies of brooding and reappraisal. The underlying assumption is that the interpersonal strategies are not mere reflections of the known intrapersonal regulation attempts but do independently predict outcome. As interpersonal processes happen in interaction not only the individual at risk for adjustment disorder was included in the models, but also the view of his or her romantic partners. Therefore, a dyadic framework assessing self-and partner-reports of interpersonal emotion regulation was applied following a state of the art framework of dyadic data analysis [31].

## Method

### Participants and procedure

The study was approved by the ethics committee of the School of Humanities and Social Sciences at the University of Zurich. Couples were recruited with online advertisements and mailing lists at the University of Zurich. The inclusion criteria were being in a committed romantic relationship and the readiness of both partners to answer the questionnaire. The study is part of a larger project investigating the mental health and regulatory processes of the couples [32]. The online questionnaire tool was programmed by cloudsolution.net. Both romantic partners registered together online, each providing an e-mail address for the invitation e-mail and informed consent. They were then invited separately by e-mail to answer an online questionnaire that took approximately 30 min to complete. This procedure allowed unequivocal matching of the couples and anonymity of the data. If the participants reported that they had a stressful event in the last 12 months and were still suffering from the effects, adjustment disorder symptoms were assessed with the Adjustment Disorder New Module ([ADNM]; see measures section). A follow-up questionnaire that is not part of the current study was sent out 3 months later. The couples were instructed not to exchange information about the questionnaires with their partners in order to avoid mutual influences. In total, 227 couples registered for the online-study. Of those 227 couples, 76 males (mean age 29.62) and 76 females (mean age 27.93) reported a stressful event in the last 12 months that still had an impact on their wellbeing. In 39 cases, both partners of one couple reported a stressful event; this was controlled for in the analysis. In three couples only, both partners reported couple conflicts as the stressor; usually, different stressors were reported. Two more females reported stressful events but had to be excluded from the analysis; as the APIM was conducted for distinguishable dyads (men and women; [31]), two same-sex couples could not be included. The participants reported the type stressful of event that occurred. For males, work stress was most often mentioned (*N* = 16), followed by time pressure (*N* = 9), and relationship conflict (*N* = 7). Female participants reported conflicts with partners (*N* = 15), conflicts with others (*N* = 7), and illness of people close to them (*N* = 7) as the three most common stressful events. Other stress events mentioned were financial problems, the death of a close relative or friend, and personal health problems. The average relationship duration was *M*_males_ = 5.32 and *M*_females_ = 5.8 years; 12 female partners and 15 male partners reported having children; and about half of the sample was cohabiting (*N*_male_ = 34; *N*_female_ = 40). Most participants were high-school graduates (Abitur, Matura as highest education degree, *N*_male_ = 15, *N*_female_ = 25); *N*_male_ = 39 and *N*_female_ = 33 participants reported having additionally a university degree. About half of the sample were students (*N*_male_ = 30, *N*_female_ = 36). The stressed sample did not differ significantly from the rest of the sample that did not report a stressful event in terms of the above-mentioned aspects.

## Measures

### Adjustment disorder new module

The ADNM questionnaire [33] assesses adjustment disorder symptoms following the stress–response concept of adjustment disorder, which will be reassembled in the proposed criteria for adjustment disorder in the ICD-11 [13]. In this study, the ADNM questionnaire was only presented when a stressful event was reported as happening in the last 12 months. The ICD-11 proposes two core symptom groups in adjustment disorder: preoccupation and failure to adapt. The preoccupation scale reflects unwanted repetitive negative thoughts about the stressor in question and includes four items (example item: “I have to think about the stressful situation a lot and this is a great burden to me”). Failure to adapt includes problems with daily functioning that started after the stressor and is assessed with four items (example item: “Since the stressful situation, I don’t like going to work or carrying out the necessary tasks of everyday life”). The items were rated on a four-point Likert scale ranging from 1 (strongly disagree) to 4 (strongly agree). Both scales yielded good internal consistency in this sample (preoccupation: females, α = .77 and males, α = .75; maladjustment: females, α = .74 and males, α = .75). The items of the scale as well as of the other measures used in this study can be found in the Additional file 1.

### Center for epidemiological studies-depression (CES-D)

This questionnaire is an established measure of depressive symptoms in general populations [34]. In the current study, the German version [35] was used to assess symptom severity as experienced in the 2 weeks prior to the beginning of the study. The 20 items are rated on a four-point Likert scale ranging from “seldom” (0) to “most of the time” (3). The total score reaches from minimum = 0 up to maximum = 60. A score of 23 is meant to indicate clinically significant levels of depressive symptoms. The scale is psychometrically well-validated and is an established instrument measuring depression that is used in different areas, including epidemiological research involving normal populations.

### Interpersonal emotion regulation: co-brooding and co-reappraisal

Co-reappraisal and co-brooding was assessed with a new instrument of interpersonal emotion regulation in couples. It refers to everyday behavior in the relationship in the last months and is assessed with a five-point Likert scale ranging from “applies not at all” (0) to “applies fully” (4). The co-reappraisal scale is the averaged score of the following items: “When I am in a bad mood, I talk with my partner … to get a new perspective on things/in order listen to the perspective of my partner to see things in a different light”. Co-brooding was assessed with these items: “When I am in bad mood, …we get stuck and circle around the reasons for my mood, and I do not feel understood by my partner/… I tell my partner the same things that bother me over and over again, even though I know that this does not make a difference/I catch myself complaining about the same things over and over again without getting responsive reactions from my partner”). The items reflect the theoretical assumption that verbalized brooding in its most maladaptive form impairs the possibility of the partner to react responsively. This is also assessed from the partner’s perspective: Both partners in the couple reported not only their own co-reappraisal and co-brooding but also perceived partner co-reappraisal and co-brooding (“When my partner is in a bad mood, … he/she talks about the same things over and over again, and I have trouble understanding her/him; … she/he is talking about the same over and over again without being open to my comments;… is it all about his/her problems and worries, I can’t do too much about it”). The inclusion of the partner perspective was meant to reduce self-report biases and to strengthen the validity in the sense of a multi-trait, multi-method approach [36] as recent views on personality assessment underline the validity of reports by informants [37]. It has been suggested to correlate aggregated means of self-ratings and informant ratings and interpret them as an accuracy score [38]. In this study, co-brooding accuracy scores were higher than co-reappraisal scores (co-brooding r_female self.male partner_ = .41**; r_male self.female partner_ = .32**; co-reappraisal r_female self.male partner_ = .17*, r_male self.female partner_ = .03). This is also reflected in the reliability measure outcomes: the Cronbach’s alpha for the co-brooding scale, including both self and partner reports, was α = .74 for females and α = .70 for males. The composite self/partner report co-reappraisal score yielded unsatisfactory results (females, α = .63; males, α = .53). Theoretically, one might argue that in contrast to co-brooding, co-reappraisal is less associated with overt, well observable behavior because it reflects the motive to change the perspective that does not necessarily need to be verbalized. Therefore, for the co-reappraisal scores, only the mean score of both self-reported items was used. This scale yielded good internal consistency in this sample (females, α = .82; males, α = .76). The items of this new scale are listed in the Additional file 1.

### Response style questionnaire: ruminative brooding

Ruminative brooding was assessed with the German version of the Response Style Questionnaire (RSQ; [39]). A previous study yielded good psychometric results for the two subscales of brooding (maladaptive) and reflection (adaptive) in a confirmatory factor analysis and tests of internal consistency and re-test reliability [40]. Accordingly, in this study the internal consistency of the subscale was high (females, α = .74; males, α = .73).

### Emotion regulation questionnaire: reappraisal

Reappraisal is a subscale of the Emotion Regulation Questionnaire [19], an often used measure of emotion regulation with good psychometric qualities [21]. In this study, the German version was used [41]. The reappraisal scale consists of items like “When I want to feel less negative emotion, I change the way I’m thinking about the situation”. The answer options range from strongly disagree (1) to neutral (4) to strongly agree (7). The scale was high in internal consistency also in this sample (females, α = .82; males, α = .79).

### Data analysis

As the data structure is dyadic and mutual interdependencies are to be expected in interpersonal emotion regulation, the models follow the suggested design of regression-based APIM [31]. This allows disentangling actor and partner effects controlling for interdependencies in the couple. In our study adjustment symptoms were only assessed if the individual was at risk, i.e. a psychologically meaningful stressor was reported. Thus, the effects of interpersonal emotion regulation in both partners on the target person at risk for adjustment disorder were analyzed. In order to not violate assumptions of independence and taking into account that in some couples both partners reported stressors, the analyses were run for the female respectively the male sample separately.

In all presented hierarchical regression models, the following variables were entered in the first step as control variables: age, whether the partner reports an event as well, whether the stressful event was related to romantic relationships, whether the stressful event was in general of an interpersonal nature, and the duration of the couple’s relationship. In the second step, actor and partner co-brooding and co-reappraisal were entered as interpersonal emotion regulation strategies. In the last step, actor and partner brooding and reappraisal were entered as intrapersonal emotion regulation strategies, thus providing the opportunity to analyze whether the predictions hold even if controlled for intrapersonal strategies.

## Results

Means, standard deviations, and correlations of the study variables are presented in Table 1. When compared with each other using T-tests for dependent samples, women report higher mean scores of co-brooding, co-reappraisal, and brooding than do men; the mean reappraisal scores are not significantly different. Table 1 shows that co-brooding is bivariately associated with preoccupation, failure to adapt, and depression. In females, there are bivariate negative associations with all three symptoms and co-reappraisal, but this is not the case for the male participants in our sample. Co-brooding is not significantly associated with intrapersonal brooding. Similarly, co-reappraisal is positively though not statistically significantly associated with its intrapersonal counterpart, reappraisal. Within the couples, there is an interdependency reflected by significant bivariate correlations of depressive symptoms and co-brooding (see Table 1). In the male sample, neither co-brooding nor co-reappraisal are significantly associated with depressive symptoms of the female partner. In contrast, the co-brooding scores of the women reporting a stressful event correlate significantly with depressive symptoms (*r*_female co-brooding.male depressio*N*=_.26*) of the male partner. There are no bivariate associations of female co-reappraisal with the male partner’s symptoms.

**Table 1:** Correlations, means, and standard deviations of the variables in the study (males with stressful event N = 73, females with stressful event *N* = 73)

Measure	*1*	*2*	*3*	*4*	*5*	*6*	*7*
1. preoccupation ADNM	−	0.42**	0.38**	0.35**	0.02	0.08	−0.09
2. failure to adapt ADNM	0.59**	−	0.45**	0.36**	−0.12	0.19 (^†^)	−0.05
3. depression CES-D	0.49**	0.70**	−	0.40**	−0.11	0.38**	−0.11
4. Co-brooding IER	0.23*	0.36**	0.29*	–	−0.15	0.15	0.02
5. Co-reappraisal IER	−0.29*	−0.31**	−0.43**	−0.34*	−	−0.22	0.24
6. brooding RSQ	0.29*	0.32**	0.42**	0.12	−0.25*	−	−0.29**
7. reappraisal ERQ	−0.06	−0.11	−0.08	−0.20 (^†^)	0.17	−0.07	−
*r* male with partner	−	−	.25*	.46*	.05	.04	.01
*r* female with partner	−	−	.24*	.45*	−.01	−.14	.03
*M* (*SD*) male	7.38 (2.68)	4.14 (1.75)	12.15 (7.95)	.80 (.67)	2.79 (1.05)	9.74 (3.1)	4.17 (1.18)
*M* (*SD*) female	8.68 (3.03)	4.25 (1.73)	13 (8.57)	1.05 (.79)	3.1 (.90)	10.55 (3.25)	4.31 (1.13)

*Note*: Intercorrelations for the male sample reporting a stressful event (*N* = 73) are presented above the diagonal, and intercorrelations for the female sample reporting a stressful event (*N* = 73) are presented below the diagonal. *ADNM* adjustment disorder new module, *CES*-*D* center of epidemiological studies –depression inventory, *IER* interpersonal emotion regulation questionnaire, *RSQ* response style questionnaire, *ERQ* emotion regulation questionnaire. (^†^) *p* < .10. *p < .05. **p < .01

### Actor and partner effects on adjustment symptoms

First, hierarchical regressions will be reported. In dyadic analysis, co-variations of both partners are usually reported; thus, the correlations of the predictors are given in Table 1. Associations between the residuals will be reported in the following results section. It is noteworthy, that the correlations between the residuals are relating different samples; the male and female samples are distinct as the samples are identified by the target individual having reported a stressful event and the partner. Thus, the correlations of the residuals might be interpreted as reflecting general gender-based associations. The results of all regression analyses are given in Table 2.

**Table 2:** APIM hierarchical multiple regression analyses predicting adjustment disorder and depressive symptoms from controls, interpersonal emotion regulation, and intrapersonal emotion regulation (males reporting a stressful event: *N* = 73; females reporting a stressful event: *N* = 73)

		Males			Females		
Steps	Predictor	β	95 % CI	ΔR^2^	β	95 % CI	ΔR^2^
	Preoccupation (ADNM)						
control + interpersonal ER	own co-brooding	0.32*	[0.16, 2.37]	.13*	0.22	[−0.28, 1.95]	.1
	partner co-brooding	0.13	[–0.61, 1.68]		−0.16	[−1.99, 0.50]	
	own co-reappraisal	0.14	[−0.25, 0.99]		−0.20^†^	[−1.53, 0.16]	
	partner co-reappraisal	0.13	[-0.30, 1.04]		−0.07	[-0.91, 0.54]	
control + interpersonal ER + intrapersonal ER	own co-brooding	0.36*	[0.28, 2.56]	.06	0.27^†^	[-0.08, 2.15]	.13*
	partner co-brooding	0.07	[−0.89, 1.45]		−0.13	[−1.77, 0.62]	
	own co-reappraisal	0.11	[−0.37, 0.93]		−0.15	[−1.32, 0.33]	
	partner co-reappraisal	0.20	[−0.14, 1.27]		−0.11	[−1.03, 0.40]	
	own reappraisal (ERQ)	−0.09	[−0.77, 0.34]		0.01	[−0.59, 0.64]	
	partner reappraisal (ERQ)	−0.26*	[−1.19,−0.02]		0.11	[−0.35, 0.95]	
	own brooding (RSQ)	−0.02	[−0.24, 0.20]		0.30*	[0.06, 0.50]	
	partner brooding (RSQ)	−0.05	[−0.25, 0.17]		−0.18	[−0.51, 0.09]	
		total R^2^	.27^†^		total R^2^	.31*	
	Failure to adapt (ADNM)						
control + interpersonal ER	own co-brooding	0.31*	[0.13, 1.48]	.1^†^	0.18	[−0.25, 1.02]	.1^†^
	partner co-brooding	0.05	[−0.56, 0.83]		0.06	[−0.55, 0.87]	
	own co-reappraisal	0.01	[−0.37, 0.40]		−0.17	[−0.81, 0.16]	
	partner co-reappraisal	0.09	[−0.24, 0.58]		−0.09	[−0.56, 0.27]	
control + interpersonal ER + intrapersonal ER	own co-brooding	0.26*	[−0.03, 1.38]	.04	0.24^†^	[−0.11, 1.16]	.13*
	partner co-brooding	0.06	[−0.55, 0.89]		0.10	[−0.42, 0.95]	
	own co-reappraisal	0.05	[−0.32, 0.49]		−0.11	[−0.69, 0.26]	
	partner co-reappraisal	0.07	[−0.31, 0.56]		−0.12	[−0.60, 0.22]	
	own reappraisal (ERQ)	0.03	[−0.30, 0.38]		0.04	[−0.30, 0.41]	
	partner reappraisal (ERQ)	0.01	[−0.34, 0.38]		0.07	[−0.27, 0.48]	
	own brooding (RSQ)	0.21^†^	[−0.01, 0.25]		0.29*	[0.03, 0.28]	
	partner brooding (RSQ)	−0.06	[−0.16, 0.09]		−0.21	[−0.31, 0.03]	
		total R^2^	.35*		total R^2^	.3*	
	Depression (CES-D)						
control + interpersonal ER	own co-brooding	0.31*	[0.45, 6.87]	.08	0.06	[−2.50, 3.70]	.17*
	partner co-brooding	−0.05	[−3.92, 2.72]		0.02	[−3.23, 3.68]	
	own co-reappraisal	−0.09	[−2.47, 1.15]		−0.39**	[−6.05,−1.34]	
	partner co-reappraisal	0.00	[−1.96, 1.93]		−0.11	[−2.88, 1.14]	
control + interpersonal ER + intrapersonal ER	own co-brooding	0.24^†^	[−0.22, 5.94]	.15*	0.09	[−2.12, 4.13]	.11^†^
	partner co-brooding	−0.05	[−3.80, 2.51]		0.05	[−2.68, 4.04]	
	own co-reappraisal	0.00	[−1.76, 1.77]		−0.32*	[−5.42,−0.77]	
	partner co-reappraisal	−0.02	[−2.11, 1.70]		−0.13	[−3.06, 0.95]	
	own reappraisal (ERQ)	−0.11	[−2.21, 0.79]		0.05	[−1.37, 2.10]	
	partner reappraisal (ERQ)	−0.05	[-1.95, 1.21]		0.04	[-1.53, 2.12]	
	own brooding (RSQ)	0.35^**^	[0.31, 1.48]		0.32*	[0.22, 1.46]	
	partner brooding (RSQ)	−0.15	[-0.95, 0.18]		−0.14	[-1.29, 0.38]	
		total R^2^	.39*		total R^2^	.32*	

*Note*. Control variables included age, relationship duration, partner also reports event, interpersonal and relationship-related nature of event (binary variables yes – no). APIM: multiple actor effects (association with own emotion regulation) and partner effects (association with partner’s emotion regulation). Co-brooding and co-reappraisal measured by *IER* interpersonal emotion regulation questionnaire (Co-brooding: composite score self- and partner’s perception), *RSQ* response style questionnaire, *ERQ* emotion regulation questionnaire, *ADNM* adjustment disorder new module, *CES*-*D* center for epidemiological studies- depression inventory

^†^ *p* ≤ .1, *p < .05, **p < .001

### Preoccupation

As reported in Table 2, the variance of preoccupation with the stressful experience is explained 27 % in the male sample (*p* = .09) and 31 % in the female sample (*p* = .04) by intra- and interpersonal emotion regulation. In the male sample, co-brooding remains a significant predictor even when controlling for intrapersonal brooding. In the female sample, the effect is marginally significant. In females, intrapersonal emotion regulation explains significant additional variance in addition to the interpersonal strategies, which is not the case with men. An important predictor in the female sample is intrapersonal ruminative brooding. In general, there are no partner effects to observe on female participants. In contrast, in the male sample, there is a significant partner effect of intrapersonal reappraisal scores of the female partner on preoccupation symptoms. The residuals of the model predicting female preoccupation correlated with *r*_male.female_ = .37* and with the residuals of the model predicting male symptoms. This suggests significant associations of the unexplained variance in both models.

### Failure to adapt

Failure to adapt is characterized by problems in daily functioning in association with the stressful event. Approximately 30 % of the variance in failure to adapt was significantly explained for both samples. A similar pattern as in the above models was revealed; co-brooding was a significant predictor for male symptoms with and without control for intrapersonal brooding. In females, controlling for intrapersonal strategies—which do explain significantly additional variance when added to the model—had a suppressor effect on co-brooding, which then significantly predicted failure to adapt. In this case, intrapersonal ruminative brooding was a significant predictor for both samples. No partner effects were observable; only interpersonal strategies explained 10 % of the variance. Residuals between the two models correlated with *r*_male.female_ = .07; this was not significant, suggesting different unexplained patterns between men and women.

### Depression

In total, more than 30 % percent of the variance (male sample, R^2^ = .39*; female sample, R^2^=. 32*) was explained by an individual’s own and their partner’s emotion regulation. Interpersonal emotion regulation and control variables explained significant amounts of variance (male sample, R^2^ = .24, *p* = .1; female sample, R^2^ = .2, *p* = .02). Additionally, an R^2^ change was at least marginally significant when adding intrapersonal emotion regulation to the models (see Table 2). An individual’s own co-brooding was significant in the male sample, while in the female sample an individual’s own co-reappraisal was a significant predictor of less depressive symptoms. An individual’s own intrapersonal ruminative brooding was a significant predictor of depressive symptoms in both samples. Residuals of both models correlated with *r*_male.female_ = .52*.

## Discussion

The aim of this study was to determine whether two newly introduced interpersonal emotion regulation strategies in couples predict adjustment symptoms above and beyond established intrapersonal emotion regulation. Furthermore, the dyadic data set allowed for the exploration of possible partner effects of intra- and interpersonal emotion regulation. We investigated three different symptoms of maladjustment: preoccupation (i.e., unwanted repetitive negative thoughts about the stressor); failure to adapt (i.e., problems in daily functioning in response to the stressor), and depressive symptoms.

In general, the results underline the importance of intra-and interpersonal emotion regulation for predicting adjustment symptoms. The beta weights suggest medium effect sizes. Co-brooding—the unwanted repetitive disclosing of negative content to the partner—was a significant predictor of symptoms above and beyond intrapersonal brooding, which was also significantly associated with symptoms. Subtle gender differences could be observed here. In the male sample, co-brooding was significant in all three symptom domains. In contrast, in the female sample co-brooding was only significant above and beyond the other strategies predicting symptoms related to daily functioning (failure to adapt) and for depressive symptoms if controlled for intrapersonal brooding. It is important to note that bivariate correlations of co-brooding with the symptom groups were also significant for women in our sample. However, controlling for an individual’s own co-reappraisal and partner co-reappraisal seemed to be relevant in this case suggesting shared variance.

Co-reappraisal—the attempt to reframe the situation cognitively in conversation with the partner—was associated with less depressive symptoms in the female sample, which was not the case in the male sample. Furthermore, it was predictive for female preoccupation, however only if the effect was not controlled for intrapersonal ruminative brooding. This suggests more overlapping variance of both interpersonal strategies and intrapersonal ruminative brooding in women, which is also reflected in significant bivariate correlation coefficients (see Table 1). In contrast, intra- and interpersonal brooding did not correlate in the male sample; nor did the two interpersonal strategies of co-brooding and co-reappraisal.

Moreover, in this sample, intrapersonal reappraisal was not associated with an individual’s own symptoms or their partner’s symptoms. However, there was one exception. Only one partner effect could be observed; if the female partner reported higher levels of reappraisal, male participants reported less preoccupation. It is important to note that this is controlled for an individual’s own reappraisal and interdependencies in the couple; thus, the partner’s tendency to reappraise was additionally associated with being less preoccupied about the stressful event above and beyond an individual’s own strategies.

In summary, it can be stated that even controlling for intrapersonal strategies, the presented measures of co-brooding and to a lesser extent co-reappraisal are emotion regulation strategies in interactions that are associated with adjustment symptoms and are not mere reflections of intrapersonal processes. The interactive nature of the regulation strategies seems to capture unique variance when it comes to explaining adjustment symptoms after a stressful event. In particular, co-brooding as the unwanted repetitive sharing of negative content with the partner seems to be highly associated with symptoms, especially in male participants. It is important to note, that this relies on a composite score of co-brooding combining the perspectives of both partners. So if both partners report this kind of interactions in the couple this is reflecting a maladaptive way of dealing together with negative content.

Further longitudinal prospective research is needed to explore whether co-brooding actually represents a pre-existing background risk factor that predicts the development of symptoms over time. The results of this study could also be interpreted in such a way that if (male) partners rely on co-brooding in the couple as an interpersonal emotion regulation strategy, it is an epiphenomenon of high symptom levels. Similar discussions have been taking place in the field of intrapersonal ruminative brooding, leading to mixed results [42]. Theoretically, rumination is expected to represent a risk factor that prolongs and intensifies depressive symptoms, maintains clinical episodes of depression, and increases the likelihood of a new episode [23]. With due caution in terms of cross-sectional data interpretation, the results support the view of interpersonal co-brooding as possibly intensifying depressed mood and adjustment disorder symptoms. Co-brooding thus seems to be relevant in the clinical presentation of adults adjusting to a stressful event and deserves further research. Recent research on intrapersonal repetitive negative thoughts underlines the potential stress-inflating and thus health-harming effect of being stuck in ruminative cycles and worries also pointing on the documented effects on physiological functioning [43]. Co-brooding could in this context be seen as doubly harmful, as it not only undermines individual coping attempts but also includes interpersonal processes that possibly reduce relationship quality. Relationship quality in turn is known to be an important factor in mental and even physical health; a recent study showed over a period of 10 years significant associations between perceived responsiveness and a physiological correlate of stress, the cortisol level [8]. Interestingly enough, the associations were mediated by negative affect, which supports the socio-affective pathway hypothesis of interpersonal emotion regulation [7]. In this study, the measure of co-brooding already included the theoretically expected reduction of relationship quality. Further research is needed to get a better understanding of the different pathways of co-brooding on the intra- and interpersonal level.

In the literature, there is evidence that adaptive emotion regulation strategies, at least as measured by retrospective self-reports, tend to have a lower degree of association with mental health outcomes than maladaptive strategies [21]. This can also be observed in this study; only a partner effect of reappraisal could be observed in addition to actor effects of co-reappraisal in women. It has been suggested that the lack of predictive power of adaptive strategies is due to more contextual variability of adaptive strategies as opposed to maladaptive strategies [22]. Recent research on reappraisal underlines this notion; in certain situations, reappraisal is not the most adaptive regulation strategy, like, for example, late in the emotion generation process when the intensity of the affective state is very high [44]. The trait-like measurement of reappraisal thus might be problematic as the fit between regulation strategy and context is neglected. This might be particularly true in the context of stress response after severe stressful experiences that tend to induce intense emotions. Further research, including taking within-person variability in different contexts into account, is needed.

When controlling for interdependencies in the couple, there are almost no partner effects. Against the possible expectation that interactive emotion regulation undertaken with the partner should show more partner effects, the only observed partner effect is that of intrapersonal reappraisal of the female partner. This is in line with the notion that when adequate social support and co-regulation is needed, an individual’s own regulation resources are of great importance [45]. Empathic reactions include empathic sharing of the affective state of the interaction partner; these reactions challenge the emotion regulation resources of the partner as well. They cannot be regulated in a functional way, and the listener will have difficulties showing empathic concern and providing responsive and supportive reactions (see [46] for a discussion of the neural basis of these processes). Therefore, the results could possibly be interpreted as pointing to the importance of adaptive emotion regulation in the co-regulating partner when it comes to coping together with stressful events. One could argue that well-regulated partners manage best the challenge of sharing empathically negative affect without suffering too much contagion of negative mood with the risk negative reciprocity. Furthermore, first studies hint to the relevance of considering an interplay of intra- and interpersonal emotion regulation strategies [47].

We found gender differences in this study; for example, women’s intra- and interpersonal emotion regulation strategies were more interrelated compared to those of the male sample. Furthermore, co-reappraisal played a more important role for women, while it was not of significance for men. In the literature, sex differences in coping have been extensively reported; for example, LK Tamres, D Janicki and VS Helgeson [48] concluded in their meta-analysis that the most pronounced sex effect was that women rely more on coping strategies which include verbal expressions to others or the self. An example of these strategies is rumination, which supposedly leads to chronic strain and has been theoretically introduced as a typical “female” phenomenon [49]. In the context of the stress-generation hypothesis in depression, these vicious circles have been interpreted as typically being associated with being female, with dispositional differences, and with role constraints [50]. This tendency to verbalize stress suggests that women rely more on interpersonal emotion regulation that do men; as expected, our data revealed baseline differences in interpersonal strategies and brooding. The amount of disclosure of personal content is very different in relationships; typically, women disclose more [51]. Co-reappraisal reflects the motive for cognitive change in the disclosure process; this might influence the quality of female disclosure in the couple relationship in a way that makes it more accessible for the male partner. This in turn might be associated with more responsive reactions by the male partner. Earlier studies show that women are more susceptible to perceived responsiveness [52] and criticism [53] in the relationship. It would be interesting to investigate this pathway in further explorations of co-reappraisal. However, in general, our data did not show profound sex differences regarding interpersonal emotion regulation, and there were no harmful partner effects on women or on men.

This study has certain limitations that must be noted: the sample is a convenience online sample and stressors as well as symptoms are self-reported. Even though doubt about how representative online studies can possibly be can be dispelled [54], it would be interesting to recruit a clinical sample and include a clinical assessment of adjustment disorder in future studies on interpersonal emotion regulation. Furthermore, these are cross-sectional data with all their limitations. However, as the research area is relatively new, the results of this cross-sectional study might encourage more elaborate studies. The use of the composite measure of co-brooding that includes both views on the process—the perspective of the individual and the partner’s perspective—might strengthen the results as, for example, the effects of social desirability should be reduced and common critic on self-report of couple processes addressed. Interestingly enough, while adding up self- and partner reports of co-brooding led to a satisfying internal consistency suggesting that both partners’ views were highly interrelated, there was a significant rater discrepancy in terms of co-reappraisal.

## Conclusions

The interpersonal view on emotion regulation in the context of stress-response seems to be supported in the current study. Adjustment disorder symptoms sensu ICD-11 are associated with interpersonal emotion regulation strategies above and beyond the links with the established common emotion regulation strategies that only look at intrapersonal processes. This hints to an added value of the investigated interpersonal strategies of emotion regulation. Being stuck in the sharing of the same negative content with the partner again and again, i.e. co-brooding, seems to represent a particularly maladaptive way of processing the stress-response for both sexes. In contrast, the tendency to collaboratively look for new, functional ways of appraising the situation (co-reappraisal) might be seen as the interactive sister of intra-personal reappraisal. This strategy seems to be adaptive, particularly for women.

As “social animals”, individuals tend to rely on social resources when trying to cope with challenging life events, and this is relevant for the regulation of emotional responses. Beside the function of regulating emotions, interpersonal strategies have an impact on relationships—for better or for worse. They have the potential to improve relationship quality and its positive correlates, but they also might be problematic for the relationship as well as the individual. In view of the above, the acknowledgement of the social context for our view of adjustment symptoms and its prevention and treatment might be a promising endeavor.

## Additional file

Additional file 1: Questionnaires. Items of the questionnaires used in this study in English. (DOCX 52 kb)

## Abbreviations

ADNM: Adjustment disorder new module
CES-D: Center for epidemiological studies-depression scale
ERQ: Emotion regulation questionnaire
ICD-11: International classification of diseases-11
RSQ: Response style questionnaire

## References

[1] Rime B, Gross JJ (2007). Interpersonal Emotion Regulation. Handbook of emotion regulation.

[2] Butler EA, Randall AK (2012). Emotional Coregulation in Close Relationships. Emot Rev.

[3] Beckes L, Coan JA (2011). Social baseline theory: The role of social proximity in emotion and economy of action. Soc Personal Psychol Compass.

[4] Coan JA, Sbarra DA (2015). Social baseline theory: The social regulation of risk and effort. Curr Opin Psychol.

[5] Niven K, Totterdell P, Holman D (2009). A classification of controlled interpersonal affect regulation strategies. Emotion.

[6] Horn AB. Interacting affect: the role of psychological intimacy for interpersonal emotion regulation under review. 2016.

[7] Debrot A, Schoebi D, Perrez M, Horn AB (2013). Touch as an interpersonal emotion regulation process in couples’ daily lives: the mediating role of psychological intimacy. Pers Soc Psychol Bull.

[8] Slatcher RB, Selcuk E, Ong AD (2015). Perceived Partner Responsiveness Predicts Diurnal Cortisol Profiles 10 Years Later. Psychol Sci.

[9] Zaki J, Williams WC (2013). Interpersonal emotion regulation. Emotion.

[10] Marroquín B (2011). Interpersonal emotion regulation as a mechanism of social support in depression. Clin Psychol Rev.

[11] Hofmann SG (2014). Interpersonal emotion regulation model of mood and anxiety disorders. Cogn Ther Res.

[12] Maercker A, Horn AB (2013). A socio-interpersonal perspective on PTSD: The case for environments and interpersonal Processes. Clin Psychol Psychother.

[13] Maercker A, Brewin CR, Bryant RA, Cloitre M, Van Ommeren M, Jones LM, Humayan A, Kagee A, Llosa AE, Rousseau C (2013). Diagnosis and classification of disorders specifically associated with stress: proposals for ICD-11. World Psychiatry.

[14] Gross JJ, Thompson RA (2007). Emotion Regulation: Conceptual Foundations. Handbook of emotion regulation.

[15] Bolger N, Zuckerman A, Kessler RC (2000). Invisible Support and Adjustment to Stress. J Pers Soc Psychol.

[16] Sbarra DA, Hazan C (2008). Coregulation, dysregulation, self-regulation: An integrative analysis and empirical agenda for understanding adult attachment, separation, loss, and recovery. Personal Soc Psychol Rev.

[17] Reis HT, Clark MS, Holmes JG (2004). Perceived Partner Responsiveness as an Organizing Construct in the Study of Intimacy and Closeness. Handbook of closeness and intimacy.

[18] Aldao A, Nolen-Hoeksema S (2010). Specificity of cognitive emotion regulation strategies: A transdiagnostic examination. Behav Res Ther.

[19] Gross JJ, John OP (2003). Individual differences in two emotion regulation processes: Implications for affect, relationships, and well-being. J Pers Soc Psychol.

[20] Webb TL, Miles E, Sheeran P (2012). Dealing with feeling: A meta-analysis of the effectiveness of strategies derived from the process model of emotion regulation. Psychol Bull.

[21] Aldao A, Nolen-Hoeksema S, Schweizer S (2010). Emotion-regulation strategies across psychopathology: A meta-analytic review. Clin Psychol Rev.

[22] Aldao A, Nolen-Hoeksema S. The influence of context on the implementation of adaptive emotion regulation strategies. Behav Res Ther. 2012. 10.1016/j.brat.2012.04.004 22659159

[23] Nolen-Hoeksema S, Morrow J (1991). A Prospective Study of Depression and Posttraumatic Stress Symptoms After a Natural Disaster: The 1989 Loma Prieta Earthquake. J Pers Soc Psychol.

[24] Nolen-Hoeksema S, Wisco BE, Lyubomirsky S (2008). Rethinking rumination. Perspect Psychol Sci.

[25] Treynor W, Gonzalez R, Nolen-hoeksema S (2003). Rumination Reconsidered: A Psychometric Analysis. Cogn Ther Res.

[26] Stroebe M, Boelen P, van den Hout M, Stroebe W, Salemink E, van den Bout J (2007). Ruminative coping as avoidance. Eur Arch Psychiatry Clin Neurosci.

[27] Rose AJ (2002). Co–Rumination in the Friendships of Girls and Boys. Child Dev.

[28] Stone LB, Hankin BL, Gibb BE, Abela JRZ (2011). Co-rumination predicts the onset of depressive disorders during adolescence. J Abnorm Psychol.

[29] Bastin M, Bijttebier P, Raes F, Vasey MW (2014). Brooding and reflecting in an interpersonal context. Personal Individ Differ.

[30] Reis HT, Shaver P (1988). Intimacy as an interpersonal process. Handbook of personal relationships: Theory, research and interventions.

[31] Kenny DA, Kashy DA, Cook WL (2006). Dyadic data analysis.

[32] Horn AB, Maercker A (2015). Anpassung an ein belastendes Ereignis im Paar: Depressionen beim Partner als Risiko fur das Auftreten von Anpassungsstorungen. Psychother Psychosom Med Psychol.

[33] Bley S, Einsle F, Maercker A, Weidner K, Joraschky P (2008). Evaluation of a new concept for diagnosing adjustment disorders in a psychosomatic setting. Psychother Psychosom Med Psychol.

[34] Radloff LS (1977). The CES-D Scale: A self-report depression scale for research in the general population. Appl Psychol Meas.

[35] Hautzinger M, Bailer M (1993). Allgemeine Depressions-Skala (ADS) [German version of the Center for Epidemiological Studies Depression Scale (CES-D)].

[36] Campbell DT, Fiske DW (1959). Convergent and discriminant validation by the multitrait-multimethod matrix. Psychol Bull.

[37] Funder DC (2001). Personality. Annu Rev Psychol.

[38] Gosling SD, Ko SJ, Mannarelli T, Morris ME (2002). A room with a cue: Personality judgments based on offices and bedrooms. J Pers Soc Psychol.

[39] Butler LD, Nolen Hoeksema S (1994). Gender Differences in Responses to Depressed Mood in a College Sample. Sex Roles.

[40] Huffziger S, Kühner C (2015). Die Ruminationsfacetten Brooding und Reflection: Eine psychometrische Evaluation der deutschsprachigen Version RSQ-10D. Z Klin Psychol Psychother.

[41] Abler B, Kessler H (2009). Emotion Regulation Questionnaire – Eine deutschsprachige Fassung des ERQ von Gross und John. Diagnostica.

[42] Bagby RM, Rector NA, Bacchiochi JR, McBride C (2004). The Stability of the Response Styles Questionnaire Rumination Scale in a Sample of Patients With Major Depression. Cogn Ther Res.

[43] Brosschot JF, Gerin W, Thayer JF (2006). The perseverative cognition hypothesis: A review of worry, prolonged stress-related physiological activation, and health. J Psychosom Res.

[44] Sheppes G, Scheibe S, Suri G, Gross JJ (2011). Emotion-regulation choice. Psychol Sci.

[45] Maisel NC, Gable SL (2009). The paradox of received social support The importance of responsiveness. Psychol Sci.

[46] Decety J, Jackson PL (2006). A social-neuroscience perspective on empathy. Curr Dir Psychol Sci.

[47] Debrot A, Schoebi D, Perrez M, Horn AB (2014). Stroking Your Beloved One’s White Bear: Responsive Touch by the Romantic Partner Buffers the Negative Effect of Thought Suppression on Daily Mood. J Soc Clin Psychol.

[48] Tamres LK, Janicki D, Helgeson VS (2002). Sex Differences in Coping Behavior: A Meta-Analytic Review and an Examination of Relative Coping. Personal Soc Psychol Rev.

[49] Nolen-Hoeksema S, Larson J, Grayson C (1999). Explaining the Gender Difference in Depressive Symptoms. J Pers Soc Psychol.

[50] Hammen C (2003). Interpersonal stress and depression in women. J Affect Disord.

[51] Dindia K, Allen M (1992). Sex differences in self-disclosure: A meta-analysis. Psychol Bull.

[52] Laurenceau J-P (2000). The interpersonal process model of intimacy in marriage: A daily-diary approach.

[53] Peterson KM, Smith DA (2010). An Actor-Partner Interdependence Model of Spousal Criticism and Depression. J Abnorm Psychol.

[54] Gosling SD, Vazire S, Srivastava S, John O (2004). Should we trust web-based studies? A comparative analysis of six preconceptions about Internet questionnaires. Am Psychol.

