# Supplementary material for: Intra- and interpersonal emotion regulation and adjustment symptoms in couples: The role of co-brooding and co-reappraisal
Source: BMC Psychol. 2016 Oct 28;4:51. doi: 10.1186/s40359-016-0159-7 (PMC5084345; doi:10.1186/s40359-016-0159-7)
Supplement: Additional file 1: — Questionnaires. Items of the questionnaires used in this study in English. (DOCX 52 kb) [file 40359_2016_159_MOESM1_ESM.docx]

Appendix: Horn & Maercker: Intra- and Interpersonal Emotion Regulation and Adjustment symptoms in Couples: The Role of Co-brooding and Co-reappraisal

**Measures (English version)**

**1) Adjustment Disorder New Module** Self-report for the assessment of adjustment disorder

**Adjustment Disorder – New Module 20**

Below is a list of stressful life events. Please indicate those events that happened during the past 1 year and are currently a very strong burden to you, or have burdened you in the last six months. You can indicate as many events as applicable. If there was no particular life event please proceed to the next questionnaire.

| Nr. |  | Yes |
| --- | --- | --- |
| 1. 1 | Relationship conflicts | □ |
|  | Conflicts with other person | □ |
|  | If yes: Whom? |  |
|  | Divorce/ separation from earlier partner | □ |
|  | Illness of a loved one | □ |
|  | Death of a loved one | □ |
|  | If yes: Who has died? |  |
|  | Problems in worklife | □ |
|  | Unemployment | □ |
|  | Pressure to meet deadlines / time pressure | □ |
|  | Financial problems | □ |
|  | Own serious illness / new episode of own illness | □ |
|  | If yes: Which illness? |  |
|  | Any other stressful event (please indicate) | □ |
|  | Any other stressful event (please indicate) | □ |

Which of the mentioned events is the most important and is currently a strain or has been straining you in the last year? Please indicate the number

_________________________________________________________________________________________

The event you indicated above as the most important: when exactly did the event happen in the run of last year. Please indicate the approximate date.

_________________________________________________________________________________________

The events you have just indicated can have numerous consequences for our well-being and behavior. Below you will find various statements about which reactions these types of event can trigger. Please answer the questions in relation to the most important event that you just indicated:

*P -> Preoccupation scale*

*F -> Maladjustment*

|  | **Does not apply** | **Does rather not apply** | **Does rather apply** | **Does very much apply** |
| --- | --- | --- | --- | --- |
| *P* 2. I have to think about the stressful situation repeatedly. | ☐ | ☐ | ☐ | ☐ |
| *P* 4. I have to think about the stressful situation a lot and this is a great burden to me. | ☐ | ☐ | ☐ | ☐ |
| *M* 10. Since the stressful situation, I find it difficult to concentrate on certain things. | ☐ | ☐ | ☐ | ☐ |
| *P* 13. I constantly get memories of the stressful situation and can’t do anything to stop them. | ☐ | ☐ | ☐ | ☐ |
| *P* 15. My thoughts often revolve around anything related to the stressful situation. | ☐ | ☐ | ☐ | ☐ |
| *M* 17. Since the stressful situation, I do not like going to work or carrying out the necessary tasks in everyday life. | ☐ | ☐ | ☐ | ☐ |
| *M* 19. Since the stressful situation, I can no longer sleep properly. | ☐ | ☐ | ☐ | ☐ |
| *M* 20. In general, the situation is constraining significantly my social life, my leisure time and my work life and other importan domains of life. | ☐ | ☐ | ☐ | ☐ |

**2) Center for Epidemiologic Studies Depression Scale (CES-D)**

*(R -> reversed items)*

Below is a list of the ways you might have felt or behaved. Please tell me how often you have felt this way during the past week.

1 = Rarely or None of the Time (Less than 1 Day)

2 = Some or a Little of the Time (1-2 Days)

3 = Occasionally or a Moderate Amount of Time (3-4 Days)

4 = Most or All of the Time (5-7 Days)

|  | 1 Rarely or none of the time | 2 some or little of the time | 3 Ocassionally or a moderate amount of time | 4 most or all of the time |
| --- | --- | --- | --- | --- |
| 1. I was bothered by things that usually don't bother me. | ☐ | ☐ | ☐ | ☐ |
| 2. I did not feel like eating; my appetite was poor. | ☐ | ☐ | ☐ | ☐ |
| 3. I felt that I could not shake off the blues even with help from my family or friends. | ☐ | ☐ | ☐ | ☐ |
| *R* 4. I felt that I was just as good as other people. | ☐ | ☐ | ☐ | ☐ |
| 5. I had trouble keeping my mind on what I was doing. | ☐ | ☐ | ☐ | ☐ |
| 6. I felt depressed. | ☐ | ☐ | ☐ | ☐ |
| 7. I felt that everything I did was an effort. | ☐ | ☐ | ☐ | ☐ |
| *R* 8. I felt hopeful about the future. | ☐ | ☐ | ☐ | ☐ |
| 9. I thought my life had been a failure. | ☐ | ☐ | ☐ | ☐ |
| 10. I felt fearful. | ☐ | ☐ | ☐ | ☐ |
| 11. My sleep was restless. | ☐ | ☐ | ☐ | ☐ |
| *R* 12. I was happy. | ☐ | ☐ | ☐ | ☐ |
| 13. I talked less than usual. | ☐ | ☐ | ☐ | ☐ |
| 14. I felt lonely. | ☐ | ☐ | ☐ | ☐ |
| 15. People were unfriendly. | ☐ | ☐ | ☐ | ☐ |
| 16. I enjoyed life. | ☐ | ☐ | ☐ | ☐ |
| 17. I had crying spells. | ☐ | ☐ | ☐ | ☐ |
| 18. I felt sad. | ☐ | ☐ | ☐ | ☐ |
| 19. I felt that people dislike me. | ☐ | ☐ | ☐ | ☐ |
| 20. I could not get “going”. | ☐ | ☐ | ☐ | ☐ |

**3) Interpersonal emotion regulation: co-brooding and co-reappraisal in couples**

*Own and partner perspective*

**When I am in a bad mood…**

|  | Applies completely | Rather applies | Neither agree, nor disagree | Applies rather not | Applies not at all |
| --- | --- | --- | --- | --- | --- |
| *Co-Reappraisal* |  |  |  |  |  |
| …I talk with my partner to get a new perspective on things/ | ☐ | ☐ | ☐ | ☐ | ☐ |
| …I talk with my partner in order listen to the perspective of my partner to see things in a different light | ☐ | ☐ | ☐ | ☐ | ☐ |
| *Co-Brooding* |  |  |  |  |  |
| …we get stuck and circle around the reasons for my mood, and I do not feel understood by my partner | ☐ | ☐ | ☐ | ☐ | ☐ |
| … I tell my partner the same things that bother me over and over again, even though I know that this does not make a difference | ☐ | ☐ | ☐ | ☐ | ☐ |
| …I catch myself complaining about the same things over and over again without getting responsive reactions from my partner | ☐ | ☐ | ☐ | ☐ | ☐ |

Partner Version:

**When my partner is in a bad mood, …**

|  | Applies completely | Rather applies | Neither agree, nor disagree | Applies rather not | Applies not at all |
| --- | --- | --- | --- | --- | --- |
| *Co-reappraisal* |  |  |  |  |  |
| … I try together with my partner to get a new perspective on things. | ☐ | ☐ | ☐ | ☐ | ☐ |
| …I try to help my partner to see things in a different light | ☐ | ☐ | ☐ | ☐ | ☐ |
| *Co-Brooding* |  |  |  |  |  |
| …we get stuck and circle around the reasons for my mood, and often can’t understand my partner well. | ☐ | ☐ | ☐ | ☐ | ☐ |
| … my partner is talking about the same things over and over again without being open for my advice. | ☐ | ☐ | ☐ | ☐ | ☐ |
| …the focus is always on my partner’s problems, I cannot do much about it. | ☐ | ☐ | ☐ | ☐ | ☐ |

**4) Response Style Questionnaire: ruminative brooding**

People think and do many different things when they feel sad, blue, or depressed. Please tell me if you never, sometimes, often, or always think or do each one when you feel down, sad, or depressed. Please indicate what you generally do, not what you think you should do.

|  | never | sometimes | often | Always |
| --- | --- | --- | --- | --- |
| Think “What am I doing to deserve this?” | ☐ | ☐ | ☐ | ☐ |
| Think “Why do I always react this way?” | ☐ | ☐ | ☐ | ☐ |
| Think about a recent situation, wishing it had gone better | ☐ | ☐ | ☐ | ☐ |
| Think “Why do I have problems other people don’t have?” | ☐ | ☐ | ☐ | ☐ |
| Think “Why can’t I handle things better?” | ☐ | ☐ | ☐ | ☐ |

**5) Emotion Regulation Questionnaire:reappraisal**

We would like to ask you some questions about your emotional life, in particular, how you control (that is, regulate and manage) your emotions.

For each item, please answer using the following scale:

1-----------------2------------------3------------------4------------------5------------------6------------------7

strongly strongly

agree neutral disagree

|  | Strongly agree |  |  | neutral |  |  | Strongly disagree |
| --- | --- | --- | --- | --- | --- | --- | --- |
|  | 1 | 2 | 3 | 4 | 5 | 6 | 7 |
| When I want to feel more positive emotion (such as joy or amusement), I change what I’m thinking about | ☐ | ☐ | ☐ | ☐ | ☐ | ☐ | ☐ |
| When I want to feel less negative emotion (such as sadness or anger), I change what I’m thinking about. | ☐ | ☐ | ☐ | ☐ | ☐ | ☐ | ☐ |
| When I’m faced with a stressful situation, I make myself think about it in a way that helps me stay calm. | ☐ | ☐ | ☐ | ☐ | ☐ | ☐ | ☐ |
| When I want to feel more positive emotion, I change the way I’m thinking about the situation. | ☐ | ☐ | ☐ | ☐ | ☐ | ☐ | ☐ |
| I control my emotions by changing the way I think about the situation I’m in. | ☐ | ☐ | ☐ | ☐ | ☐ | ☐ | ☐ |
| When I want to feel less negative emotion, I change the way I’m thinking about the situation. | ☐ | ☐ | ☐ | ☐ | ☐ | ☐ | ☐ |
